# Signatures of selection reveal candidate genes involved in economic traits and cold acclimation in five Swedish cattle breeds

**DOI:** 10.1186/s12711-020-00571-5

**Published:** 2020-09-04

**Authors:** Seyed Mohammad Ghoreishifar, Susanne Eriksson, Anna M. Johansson, Majid Khansefid, Sima Moghaddaszadeh-Ahrabi, Nahid Parna, Pourya Davoudi, Arash Javanmard

**Affiliations:** 1 Department of Animal Science, University College of Agriculture and Natural Resources, University of Tehran, Karaj, 31587-11167 Iran; 2 Department of Animal Breeding and Genetics, Swedish University of Agricultural Sciences, SE-75007 Uppsala, Sweden; 3 AgriBio Centre for AgriBioscience, Agriculture Victoria, Bundoora, VIC 3083 Australia; 4 Department of Animal Science, Faculty of Agriculture and Natural Resources, Islamic Azad University, Tabriz Branch, Tabriz, Iran; 5 Department of Animal Science and Aquaculture, Dalhousie University, Truro, NS B2N5E3 Canada; 6 Department of Animal Science, Faculty of Agriculture, University of Tabriz, Tabriz, Iran

## Abstract

**Background:**

Thousands of years of natural and artificial selection have resulted in indigenous cattle breeds that are well-adapted to the environmental challenges of their local habitat and thereby are considered as valuable genetic resources. Understanding the genetic background of such adaptation processes can help us design effective breeding objectives to preserve local breeds and improve commercial cattle. To identify regions under putative selection, GGP HD 150 K single nucleotide polymorphism (SNP) arrays were used to genotype 106 individuals representing five Swedish breeds i.e. native to different regions and covering areas with a subarctic cold climate in the north and mountainous west, to those with a continental climate in the more densely populated south regions.

**Results:**

Five statistics were incorporated within a framework, known as de-correlated composite of multiple signals (DCMS) to detect signatures of selection. The obtained *p*-values were adjusted for multiple testing (FDR < 5%), and significant genomic regions were identified. Annotation of genes in these regions revealed various verified and novel candidate genes that are associated with a diverse range of traits, including e.g. high altitude adaptation and response to hypoxia (*DCAF8*, *PPP1R12A*, *SLC16A3*, *UCP2*, *UCP3*, *TIGAR*), cold acclimation (*AQP3*, *AQP7*, *HSPB8*), body size and stature (*PLAG1*, *KCNA6*, *NDUFA9*, *AKAP3*, *C5H12orf4*, *RAD51AP1*, *FGF6*, *TIGAR*, *CCND2*, *CSMD3*), resistance to disease and bacterial infection (*CHI3L2*, *GBP6*, *PPFIBP1*, *REP15*, *CYP4F2*, *TIGD2*, *PYURF*, *SLC10A2*, *FCHSD2*, *ARHGEF17*, *RELT*, *PRDM2*, *KDM5B*), reproduction (*PPP1R12A*, Z*FP36L2*, *CSPP1*), milk yield and components (*NPC1L1*, *NUDCD3*, *ACSS1*, *FCHSD2*), growth and feed efficiency (*TMEM68*, *TGS1*, *LYN*, *XKR4*, *FOXA2*, *GBP2*, *GBP5*, *FGD6*), and polled phenotype (*URB1*, *EVA1C*).

**Conclusions:**

We identified genomic regions that may provide background knowledge to understand the mechanisms that are involved in economic traits and adaptation to cold climate in cattle. Incorporating *p*-values of different statistics in a single DCMS framework may help select and prioritize candidate genes for further analyses.

## Background

According to the natural selection theory, favorable mutations may increase the chance of individuals to survive and reproduce, which results in an increased frequency of fitness-related alleles in a species/breed over generations [1]. Likewise, human-mediated selection may increase the frequency of favorable alleles associated with economic traits. Therefore, the combination of these two events shapes the genetic architecture of cattle breeds by leaving footprints in the genome that might be detectable. The identification of signatures of selection becomes more complicated when demographic events such as population bottleneck, admixture, genetic drift, and inbreeding have occurred. However, when selection footprints are successfully identified, they can contribute to a better understanding of the processes that cause diversity among breeds, and to pinpoint the causal variants that are involved in a phenotype under selection.

The recent development of cost-effective genotyping technologies has made it possible to scan the genome to uncover regions that are under putative selection. Similarly, different statistical tests have been conceptualized to detect signatures of selection and pinpoint the causal variants by using various models. The rationale behind selection signature theory is that the frequency of alleles that are under selection can vary in opposite directions (low or high), and thus results in stretches of consecutive homozygous genotypes [2], or in modifying the length and frequency of haplotypes around the region. Several statistical tests have been proposed to detect selection signals e.g. [3–6]. One limitation of using a single statistical test is that the variant under selection may have an effect on adjacent loci, depending on the extent of linkage disequilibrium, which can reduce the resolution of the mapping of selection signatures [7]. Different single statistic tests often do not return consistent results [8, 9]. The inconsistency among results may be attributed to factors such as different sensitivity to sampling design, details of the selective sweep, and the demographic history of populations under study [8–10]. A popular strategy to overcome these issues is to prioritize selection signals detected by several single statistical tests e.g. [11, 12]; but following this strategy, we may lose some loci that show weak signals [9]. Hence, strategies based on combining *p*-values of different test statistics (composite measures of selection) have been developed [13–15]. One of these is the de-correlated composite of multiple signals (DCMS) [15] used in this study. Ma et al. [15] reported that the resolution of selection signature mapping and the power of detecting selection signals were improved by using DCMS compared to most single statistics. Moreover, composite measures such as DCMS have been reported to identify the causal variants (i.e. the variants under selection in the detected signature regions) more precisely [7].

According to a FAO report, ~ 1000 distinct cattle breeds are listed and are geographically distributed in different countries around the world. Swedish cattle breeds (*Bos taurus*) include both indigenous/native and commercial breeds [16]. Whereas the latter are economically superior regarding production of meat and milk, the former are well adapted to the challenges imposed by their local environment [16], and show superior phenotypes for certain traits. One example is the favorable milk protein composition for cheese-making of the two Swedish mountain breeds: Fjäll and Fjällnära cattle [16, 17]. Until the 20th century, the old local breeds were of major importance in Sweden, representing several hundred thousand heads [18]. The number of individuals decreased rapidly in the early to mid-20th century due to the development of more efficient breeding programs using e.g. artificial insemination (AI), and to the increased competition from breeds with a higher milk yield [19]. However, due to the growing awareness of the value of local breeds as genetic resources, several actions have been adopted for their preservation. The native Swedish cattle breeds still display considerable phenotypic diversity in terms of coat color, body weight and size, milk content, and polled phenotype, as well as substantial genetic variation, as described by Upadhyay et al. [16]. Moreover, indigenous breeds may carry specific gene variants that contribute to adaptation to their local environment. Knowledge about the genomic regions that display signatures of selection in breeds adapted to different environments is of great value to design future breeding strategies, both in local breeds and in larger commercial breeds [20, 21].

Well-documented studies on signatures of selection have shown that they can help identify polymorphisms and/or candidate genes that underlie economical traits in cattle breeds. As reviewed by Gutiérrez-Gil et al. [22], several genes have been identified, for example *ATP binding cassette subfamily G member 2 (junior blood group)* (*ABCG2*), which is associated with milk composition, *coiled*-*coil*-*helix*-*coiled*-*coil*-*helix domain containing 7* (*CHCHD7*) and *PLAG1 zinc finger* (*PLAG1*) with body size, *diacylglycerol O*-*acyltransferase 1* (*DGAT1*) with milk production, *XK related 4* (*XKR4*) with growth trait, and *melanocortin 1 receptor* (*MC1R*) and *KIT proto*-*oncogene, receptor tyrosine kinase* (*KIT*) with coat color and spotting. Such findings are important to understand the mechanisms that explain the phenotypic diversity among breeds. However, these studies were often based on single statistical tests which have limited power to detect selection signatures. Thus, the purpose of our study was to combine multiple statistics of signatures of selection within a single DCMS framework [15] by taking the correlation between them into account to detect signatures of selection in the genome of Swedish indigenous and commercial cattle breeds with greater statistical power and higher resolution.

## Methods

### Sample collection and data quality control

Genotype data of Swedish cattle breeds obtained with GeneSeek^®^ Genomic Profiler High-Density Bovine 150 K (GGP HD150K) single nucleotide polymorphisms (SNPs) array that were previously described in a study by Upadhyay et al. [16] were downloaded from the DRYAD (https://datadryad.org/) public data repository. Five breeds including Fjällnära (n = 16), Fjäll (n = 23), Swedish Holstein–Friesian (n = 24), Swedish Red (n = 25), and Swedish Red Polled (n = 18) were retained for subsequent analyses (Table 1), whereas the remaining four breeds with a small sample size were discarded. All individuals from the five retained breeds, except one Swedish Red Polled individual born in 2016, were born between the mid-1970s and the early 2000s [16]. Inclusion of very close relatives such as full-sibs or parent–offspring–pairs was avoided as much as possible by using available information on the location of the farms and on pedigrees [16]. Detailed information about sample collection and DNA extraction is in [16]. This dataset overlapped partially with that used in the study by Johansson et al. [17].

**Table 1:** Descriptive statistics for the studied Swedish cattle breeds

Breed	N samples	Type (distribution)	Characteristic	Citation
FNC	16	Native (north)	Smaller body size than the Fjäll breed; lower milk yield; said to be hardy and hold strong ability to find food in natural pastures	[16]
SMC	23	Native (north)	Small body size of cows about 400-450 kg; said to be hardy and hold strong ability to find food in natural pastures	[16]
SHF	24	Commercial (south)	The old Friesian type was more of a dual-purpose; the SHF is a commercial dairy breed with live weight of cows around 700 kg	[16]
SRC	25	Commercial (south middle to south)	A commercial dairy breed; live weight of cows 550–650 kg	[16]
SRP	18	Native (middle)	The focus is on conservation; said to be hardy; live weight of cows 350–600 kg	[16]

Breed: FNC (Fjällnära Cattle), SMC (Fjäll also known as Swedish Mountain Cattle), SHF (Swedish Holstein–Friesian), SRC (Swedish Red Cattle), SRP (Swedish Red Polled)

N samples: number of genotyped samples (i.e. before data quality control); note that according to data quality control, four samples (due to animal call-rate < 0.95) were removed, and principal component (PC) analyses, three samples (due to locating outside their expected breed cluster) were removed; resulting in 99 individuals for analyses of selection signatures

Data quality control was performed using the PLINK v1.9 [23] software, separately for each of the two breed groups, including the northern breeds (Fjällnära and Fjäll) and middle–southern Swedish breeds (Swedish Holstein–Friesian, Swedish Red, and Swedish Red Polled) (see Table 1). SNPs with a call rate lower than 0.95 and a MAF lower than 0.05, and those for which the Hardy–Weinberg equilibrium Chi square test *p-*value was lower than 10^−6^ were discarded. In addition, SNPs that were duplicated in the map file, or located on a sex chromosome, and/or had an unidentified position on the UMD3.1 assembly [24] were removed using the PLINK --exclude option. After merging the two datasets (using PLINK’s --merge option), a subset of 105,362 mutual SNPs and 102 individuals (with an overall call rate of 99.92%) remained for subsequent analyses. It should be noted that within-group quality control was performed to provide high-quality SNPs for haplotype phasing as requested by SHAPEIT2 [25].

### Principal component (PC) analysis

We used the PLINK --ibs-matrix command to estimate the identity-by-state (IBS) matrix between individuals. The output was used to perform a principal component (PC) analysis of genetic distances with the *prcomp* R function to visualize the distribution of samples, and the results were plotted by using R (https://www.r-project.org/).

### De-correlated composite of multiple signals (DCMS)

In this study, five statistics including fixation index (F_ST_) [3], haplotype homozygosity (H1) [5], modified haplotype homozygosity (H12) [5], Tajima’s *D* index [6], and nucleotide diversity (pi) [4] were combined into a single DCMS framework [15] as described in Yurchenko et al. [20]. DCMS is similar to composite selection signals (CSS) [13] by combining *p*-values, with the difference that it takes the respective correlation between the various statistics into account [7, 15]. The DCMS statistic can be calculated at position $l$ as follows [15]:

1 $$DCMS_{l} = \mathop \sum \limits_{t = 1}^{n} \frac{{{ \log }\left[ {\frac{{1 - p_{lt} }}{{p_{lt} }}} \right]}}{{\mathop \sum \nolimits_{i = 1}^{n} \left| {r_{it} } \right|}} ,$$

where $p_{lt}$ shows the *p*-value at position $l$ for statistic $t$; $r_{it}$ refers to the correlation between the test statistic of the $i^{th}$ and $t^{th}$ methods, and $n$ is the total number of test statistics (combined) in the DCMS. The expression $1/\mathop \sum \limits_{i = 1}^{n} \left| {r_{it} } \right|$ is called weight factor, which ranges from $1/n$ to 1. For example, in a given dataset with n = 3 different uncorrelated ($r_{i \ne j} = 0$) test statistics (*t*), the weight factor will be 1 for each *t* and $DCMS_{l}$ will be the sum of the log ($\left( {1 - p_{lt} } \right)/p_{lt}$); if the three statistics are fully correlated ($r_{i \ne j} = 1$), the weight factor for each statistic (*t*) will be $1/3$, and $DCMS_{l}$ will be the average of ${ \log }(\left( {1 - p_{lt} } \right)/p_{lt}$). Indeed, the weight factors help avoiding excessive contribution of highly correlated statistics in the DCMS calculation.

## Haplotype-based H1 and H12 statistics

Effective population size (N_e_) is a required parameter in haplotype phasing. It was initially estimated, separately, for each of the two breed groups using the SNeP software [26] with the parameters described in [27]. SHAPEIT2 [25] was used for haplotype phasing of the autosomal genome, also separately for each breed group. In SHAPEIT2, we set the parameters conditioning states to 400 (--states 400), and the N_e_ to 108 (--effective-size 108) for the group of southern–middle breeds and 60 (--effective-size 60) for the group of northern breeds. A bovine genetic map [28] was used to accompany SHAPEIT2 for haplotype phasing in order to correct for the variation in recombination rate along the cattle genome. Then, using a customized R script, the phased haplotypes were transformed to the format requested by the H12_H2H1.py script (https://github.com/ngarud/SelectionHapStats). This python script with a window size of 14 SNPs and step size of 1 (-window 14 -jump 1) was run to estimate the H1 and H12 statistics for each autosome and each breed, as described in [20].

### Tajima’s D and nucleotide diversity (pi) statistics

Both Tajima’s *D* and pi statistics were estimated with the vcftools software [29]. Tajima’s *D* statistics were calculated for each breed and chromosome, separately, using the --TajimaD function considering non-overlapping sliding windows of 300 Mb (--TajimaD 300000). The estimated *D* values for each 300-Mb bin were assigned to SNPs within that bin, and missing values were converted into zeros. Pi statistics were calculated for each breed and chromosome separately with the --site-pi function; and in order to reduce noise, the outputs were smoothed for each chromosome through the R’s *runmed* function with a window size of 31 SNPs (k = 31, endrule = “constant”) as described in [20].

### Fixation index (F_ST_)

Fixation index, which is a measure of population differentiation, was calculated for each SNP and each breed against the remaining samples from the other breeds using PLINK --fst and --within functions. F_ST_ values less than 0 were converted into zeros and the statistics were then smoothed using the *runmed* function as described for the pi statistics.

### Calculation of the DCMS statistic

The genome-wide DCMS was calculated for each breed, separately, by combining the five statistics (H1, H12, Tajima’s *D*, pi, and F_ST_) for each SNP. Initially, for each statistic within a given breed, genome-wide *p*-values based on fractional ranks (i.e. the *stat_to_pvalue* function of the R MINOTAUR package [30]) were calculated for all the SNPs. To this end, a left tailed test for the pi and *D* statistics (two.tailed = FALSE, right.tailed = FALSE) and a right tailed test for the H1, H12, and F_ST_ statistics (two.tailed = FALSE, right.tailed = TRUE) were applied. Then, we called the *covNAMcd* function (alpha = 0.75, nsamp = 50,000) from the rrcovNA R package [31] to calculate an n × n correlation matrix (i.e. the minimum covariance determinant estimator of multivariate location and scatter) between the included statistics (where n represents the number of statistics to estimate the DCMS values). This matrix was used as input in the *DCMS* function of the MINOTAUR R package [30] to calculate genome-wide DCMS values. Once the DCMS values were generated, they were fitted to a normal distribution using the robust linear model (*rlm*) function of the MASS R package [32] as described in [20, 21]: model = rlm (dcms ~ 1), in which the dcms object is a vector containing the raw DCMS values. The outputs of the fitted model (i.e. Mu [mean] and SD [standard deviation]) were used as input in the *pnorm* R function to calculate the *p*-values of the DCMS statistics: dcms_pvalues = pnorm (q = dcms, mean = Mu, sd = SD, lower.tail = FALSE). In order to control multiple testing false discovery rate (FDR) among rejected null hypotheses [33], the DCMS *p*-values were transformed into the corresponding q-values according to the Benjamini and Hochberg method [34] using the *p.adjust* R function: dcms_qvalues = p.adjust (p = dcms_pvalues, method = ”BH”, n = length(dcms_pvalues)).

### Gene annotation

Genes that were located in genomic regions including consecutive SNPs with a q-value lower than 0.05 were considered as statistically significant intervals and the boundary for each interval was determined by searching for the first flanking SNP showing a q-value higher than 0.1. Then, the protein coding genes were extracted from the significant regions based on the UMD3.1 bovine reference genome assembly [24]. Finally, we performed an extensive review of the literature to annotate functions of the identified genes.

## Results

**Fig. 1:**
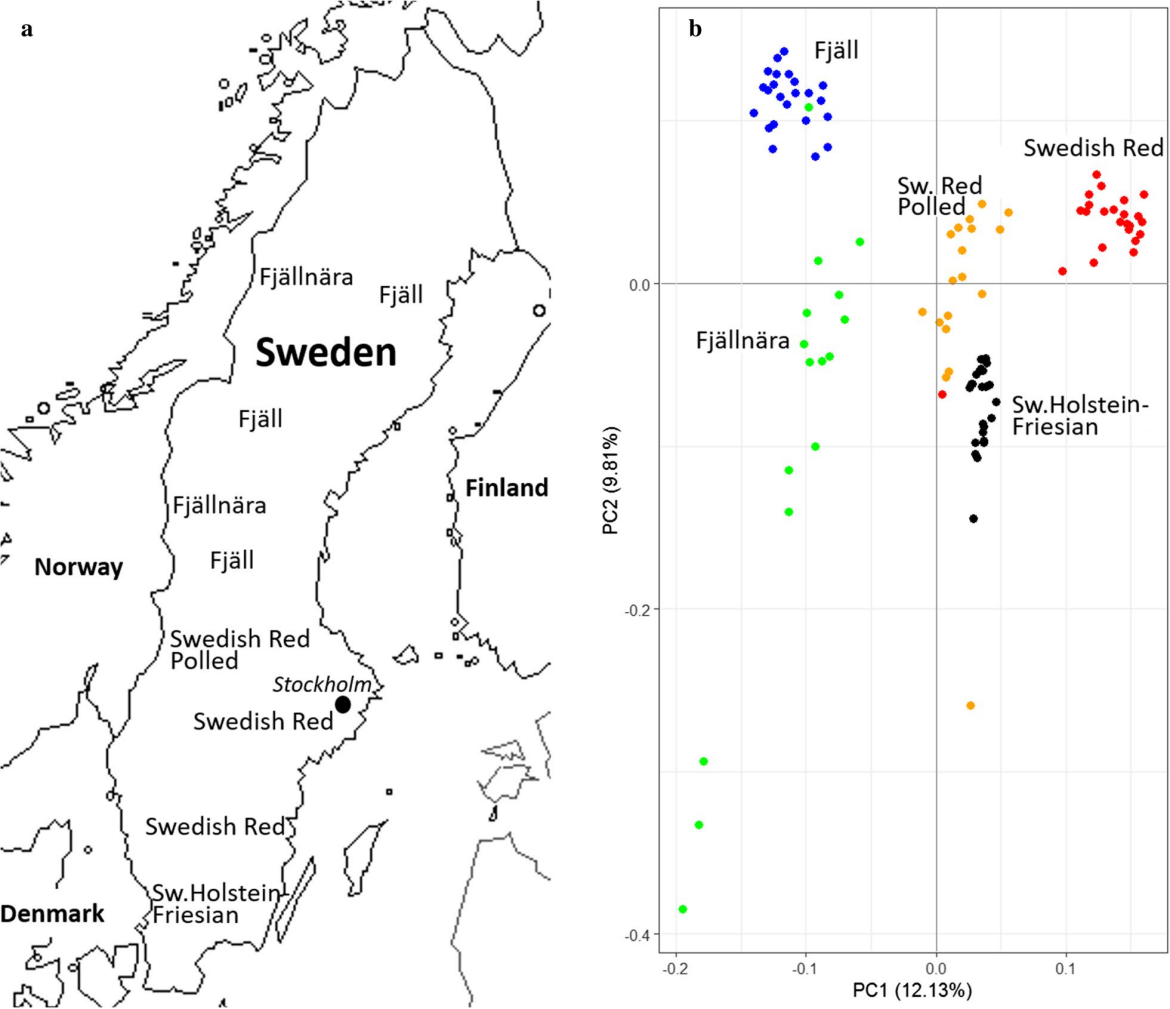
Geographic distribution (**a**) and PC analyses (**b**) of the Swedish cattle breeds used in this study. Figure 1a is adapted with permission from “Genomic relatedness and diversity of Swedish native cattle breeds” by Maulik Upadhyay et al. [16]. Copyright 2019 by Creative Commons Attribution 4.0 International License (http://creativecommons.org/licenses/by/4.0/)

As illustrated in Fig. 1a, five cattle breeds that originated from the northern, middle and southern parts of Sweden were included in our study. According to the PC analyses (Fig. 1b), the first PC clearly differentiated our dataset into two clusters of breeds, i.e. the northern breeds (Fjäll and Fjällnära) and the middle–southern breeds (Swedish Holstein–Friesian, Swedish Red Cattle, and Swedish Red Polled). PC analysis of these cattle breeds (Fig. 1b**)** led us to remove three samples that were located outside of their expected breed cluster. Therefore, 99 individuals (genotyped for 105,362 SNPs) remained for analyses of signatures of selection. Table 1 presents the characteristics and descriptive statistics for each breed. The average distance between the adjacent SNP pairs among the 105,362 SNP genotypes was 24.89 (± 21.48) kb, which represented 2.62 Gb of the UMD 3.1 bovine genome assembly. The average distance between adjacent SNP pairs ranged from 24.36 on *Bos taurus* chromosome (BTA)27 to 25.29 on BTA11 (data not shown).

### De-correlated composite of multiple signals (DCMS)

After calculation of the within-breed DCMS statistics for 105,362 SNPs, *p*-values were fitted to a normal distribution and corrected for multiple testing (FDR < 0.05). We identified 58, 37, 38, 39, and 51 genomic regions for the Fjällnära, Fjäll, Swedish Holstein–Friesian, Swedish Red, and Swedish Red Polled breeds, respectively (Table 2) and [see Additional file 1: Table S1]. The average length of the significant regions ranged from 142.3 (± 187.1) kb in Swedish Red Polled to 315.4 (± 400.8) kb in Swedish Red Cattle (Table 2). The total length of the genome covered with significant signals and the number of identified protein coding genes between breeds ranged from 72.6 to 134.1 Mb and from 61 to 99, respectively (Table 2). Overall, 355 unique protein coding genes were detected within the significant intervals for all the breeds (Table 2) and [see Additional file 2: Table S2], among which several were detected in more than one breed [see Additional file 3: Table S3].

**Table 2:** Descriptive statistics of the genomic regions identified through the de-correlated composite of multiple signals (DCMS) in the five Swedish native cattle breeds

Breed	N regions	Average ± SD (Kb)	N SNPs	Total size (Mb)	N genes
Fjällnära Cattle	58	231.1 ± 298.1	719	134.1	90
Fjäll Cattle	37	229.5 ± 226.2	421	84.9	61
Sw. Holstein–Friesian	38	249.1 ± 289.4	543	94.7	99
Sw. Red Cattle	39	315.4 ± 400.8	641	123.0	84
Sw. Red Polled	51	142.3 ± 187.1	499	72.6	61

N Regions: number of segments identified using the DCMS method as significant genomic regions harboring signatures of selection

N SNPs: number of SNPs identified within the regions detected as signatures of selection using the DCMS method (q-value < 0.05)

N genes: number of protein coding genes identified within the significant regions detected by the DCMS method

**Fig. 2:**
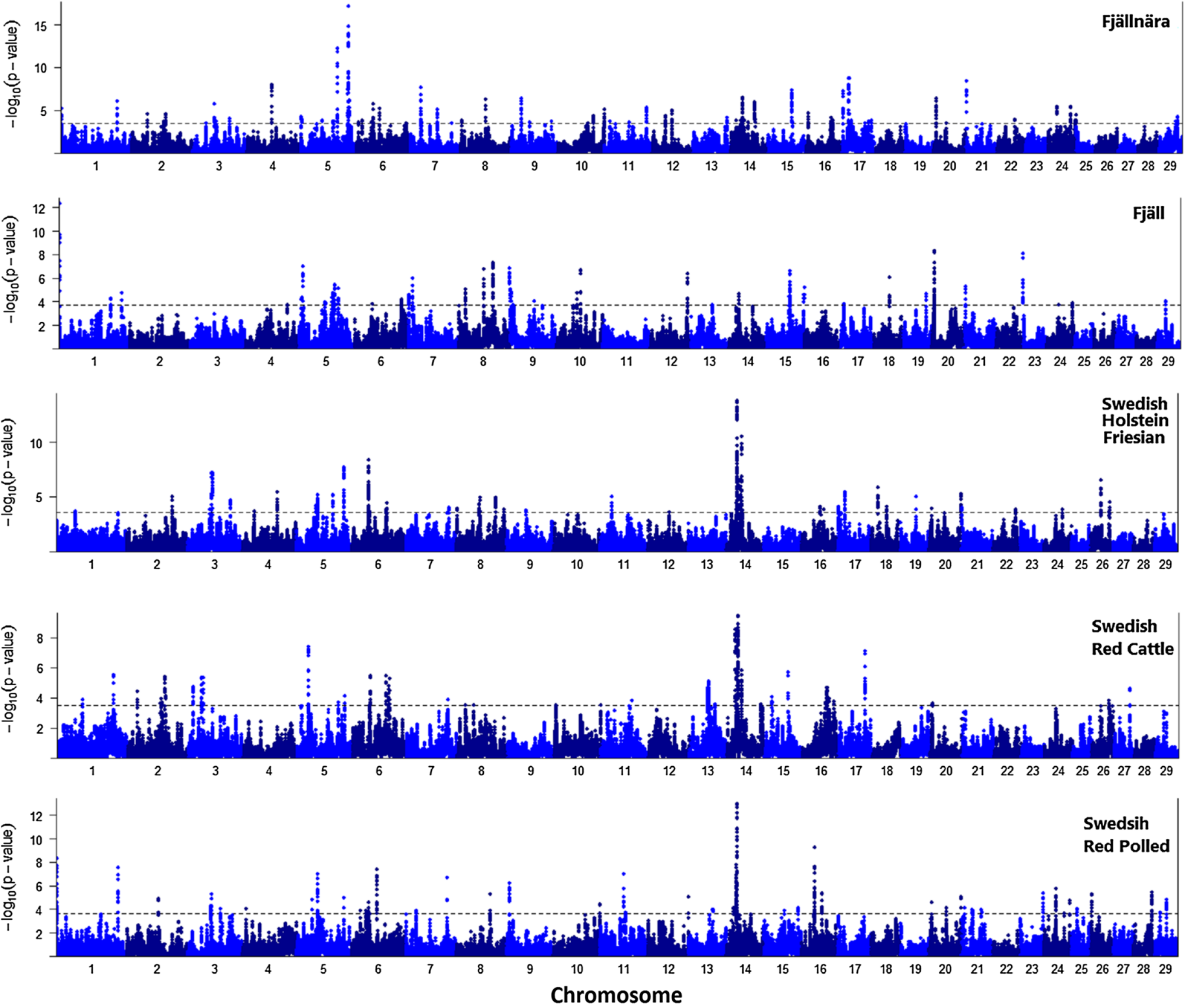
Manhattan plot of the genomic regions detected by the DCMS method as being under putative selection. The dashed lines represent the significant threshold level at a FDR of 5% (i.e. q-value < 0.05)

The distribution of the regions of signatures of selection across the genome of the five Swedish breeds is represented in Fig. 2. The most significant genomic regions were identified on BTA5 in Fjällnära (BTA5: 105,75-106,52), on BTA1 in Fjäll (BTA1: 2,25-2,52), and on BTA14 in Swedish Holstein–Friesian (BTA14: 24,42-25,11 & 14: 25,35-25,73), Swedish Red Cattle (BTA14: 24,00-24,89), and Swedish Red Polled (BTA14: 24,00-24,26) breeds (Fig. 2) and [see Additional file 1: Table S1].

Gene annotation of the identified regions detected various verified and novel candidate genes that are associated with a diverse range of traits including high altitude adaptation and response to hypoxia (*DCAF8*, *PPP1R12A*, *SLC16A3*, *UCP2*, *UCP3*, *TIGAR*), cold acclimation (*AQP3*, *AQP7*, *HSPB8*), body size and stature (*PLAG1*, *KCNA6*, *NDUFA9*, *AKAP3*, *C5H12orf4*, *RAD51AP1*, *FGF6*, *TIGAR*, *CCND2*, *CSMD3*), resistance to disease and bacterial infection (*CHI3L2*, *GBP6*, *PPFIBP1*, *REP15*, *CYP4F2*, *TIGD2*, *PYURF*, *SLC10A2*, *FCHSD2*, *ARHGEF17*, *RELT*, *PRDM2*, *KDM5B*), reproduction (*PPP1R12A*, *ZFP36L2*, *CSPP1*), milk yield and components (*NPC1L1*, *NUDCD3*, *ACSS1*, *FCHSD2*), growth and feed intake (*TMEM68*, *TGS1*, *LYN*, *XKR4*, *FOXA2*, *GBP2*, *GBP5*, *FGD6*), and polled phenotype (*URB1*, *EVA1C*) (see Table 3).

**Table 3:** Genomic autosomal regions detected (through the DCMS analyses) as being under putative selection in Swedish native and commercial cattle breeds

Region (Mb)	Q-value	Breed	Candidate gene (rank)	Trait	Citation
1: 1.58-2.39	0.00274	FNC	*EVA1C* (1), *C1H21orf59* (2), *SYNJ1* (3), *PAXBP1* (4), *C1H21orf62* (5), *OLIG1* (6), *LOC526226* (7), *IFNAR2* (8)	Polled phenotype	[105]
1: 2.25-2.52	4.9E − 08	SMC	*URB1* (1), *EVA1C* (2), *MRAP* (3), *MIS18A* (4)	Polled phenotype	[105]
1: 1.14-1.33	0.00289	SRP	*CRYZL1* (5), *DONSON* (4), *SON* (3), *GART* (2), *DNAJC28* (10)	Polled phenotype	[105]
1: 1.52-2.51	1.2E − 05	SRP	*IL10RB* (12), *IFNAR2* (11), *LOC526226* (10), *OLIG1* (9), *C1H21orf62 (8)*, *PAXBP1* (7), *SYNJ1* (6), *C1H21orf59* (5), *EVA1C* (2), *URB1* (1), *NSBTAG00000023718* (3), *MIS18A* (4)	Polled phenotype	[105]
2: 71.46-71.66	0.00633	SRP	*TMEM37* (1)	Disease resistance	[94]
3: 32.06-32.34	0.00325	SRC	*CHIA* (2), *CHI3L2* (4)	Bacterial infection	[96, 97]
3: 9.23-9.72	0.00933	SRC	*DCAF8* (1)	Altitude adaptation	[88]
3: 9.57-9.58	0.00933	SRC	*PEA15* (2)	RFI	[77]
3: 54.20-54.22	6.8E − 05	SHF	*GBP6* (4)	Bacterial infection	[103]
3: 54.34-54.36	6.87E − 05	SHF	*GBP2* (3)	RFI	[77]
3: 54.64-54.68	6.8E − 05	SHF	*GBP4* (11)	Stature	[106]
3: 54.25-54.26	6.8E − 05	SHF	*GBP5* (2)	RFI	[77]
4: 77.43-77.70	0.00202	SHF	*NPC1L1* (5), *NUDCD3* (6)	Milk yield	[107]
5: 105.75-106.52	2.60e-13	FNC	*KCNA6* (13), *NDUFA9* (11), *AKAP3* (10), *C5H12orf4* (7), *RAD51AP1* (6), FGF6 (2), *TIGAR* (3), CCND2 (4)	Body weight	[76, 77]
			*CCND2* (4)	Stature	[78]
5: 82.49-82.73	2.37e-09	FNC	*PPFIBP1* (2), *REP15* (3)	Bacterial infection	[99, 108]
5: 106.15-106.32	2.4E − 05	SHF	*FGF6* (2), *TIGAR* (3), *CCND2* (4)	Body weight	[76]
			*CCND2* (4)	Stature	[78]
5: 9.30-9.56	0.00039	SMC	*PPP1R12A* (1)	Reproduction	[109]
			*PPP1R12A* (1)	Altitude adaptation	[85, 86]
5: 24.92-25.02	8.17E − 05	SRC	*FGD6* (3)	RFI	[77]
6: 37.06-37.27	5.7E − 06	SHF	*TIGD2* (2)	Bacterial infection	[110]
6: 37.64-37.72	0.01986	SHF	*PYURF* (1)	Bacterial infection	[110]
7: 8.40-8.48	0.00159	SMC	*CYP4F2* (1)	Disease resistance	[93, 94]
8: 76.23-76.78	0.00022	SMC	*AQP3* (5), *AQP7* (5)	Acclimation	[89]
11: 25.57-25.83	0.00433	SHF	*ZFP36L2* (1)	Reproduction	[111]
12: 83.19-83.37	0.00082	SMC	*SLC10A2* (1)	Bacterial infection	[95]
13: 40.38-42.28	0.00985	SRC	*FOXA2* (6)	Growth traits	[63]
13: 43.40-43.42	0.00474	SRC	*ASB13* (9)	RFI	[77]
13: 42.73-44.04	0.00474	SRC	*ACSS1* (7)	Milk content	[112, 113]
14: 53.71-54.81	0.00068	FNC	*CSMD3* (1)	Body length	[80]
14: 24.42-25.11	2.2E − 10	SHF	*TMEM68* (2), *TGS1* (1), *LYN* (3)	Growth traits	[114]
14: 24.42-25.11	2.2E − 10	SHF	*TGS1* (1), *LYN* (3), *RPS20* (4), *MOS* (5), *PLAG1* (6), *CHCHD7* (7)	Pleiotropic	[44, 48, 50, 56, 114]
14: 25.35-25.73	2.2E − 10	SHF	*IMPAD1 (*1)	Pleiotropic	[51, 64, 66]
14: 26.01-26.24	0.00031	SHF	*FAM110B* (1)	Pleiotropic	[64]
14: 24.00-24.89	2.8E − 06	SRC	*XKR4* (1), *TMEM68* (2)	Growth, feed intake	[114]
14: 25.50-25.58	0.00624	SRC	*IMPAD1* (1)	Pleiotropic	[64, 115]
14: 33.02-33.41	0.00513	SRC	*CSPP1* (1)	Reproduction	[116]
14: 33.02-33.41	0.00513	SRC	*COPS5* (2)	Feed intake	[117]
14: 32.10-32.63	0.00137	SRC	*CRH* (1)	Growth	[60]
15: 53.10-54.25	5.6E − 05	FNC	*FCHSD2* (1)	Milk fat content	[70]
15: 53.10-54.25	5.6E − 05	FNC	*FCHSD2* (1), *ARHGEF17* (5), *RELT* (6)	Bacterial infection	[92]
			*UCP2* (13)	Hypoxia	[118]
			*UCP2* (13), *UCP3* (14)	Milk fat content, carcass quality	[71–73, 119]
15: 53.13-53.80	0.00073	SMC	*FCHSD2* (1)	Milk fat content	[70]
15: 53.13-53.80	0.00073	SMC	*FCHSD2* (1), *ARHGEF17* (4), *RELT* (5)	Bacterial infection	[92]
15: 53.52-53.67	0.00178	SRC	*ARHGEF17* (1), *RELT* (2)	Bacterial infection	[92]
15: 78.33-78.51	0.02265	SRP	*SLC39A13* (1)	Stature	[120, 121]
16: 55.09-55.15	0.00963	SRC	*PRDM2* (1)	Bacterial infection	[77, 99]
			*PRDM2* (1), *KDM5B* (2)	Bacterial infection	[98, 99]
			*PRDM2* (1)	RFI	[77]
16: 44.96-45.25	0.00299	SRP	*SPSB1* (1)	Immune regulation	[100, 101]
17: 57.91-58.43	0.00011	SRC	*HSPB8* (1)	Heat and cold stress	[122, 123]
19: 51.21-51.30	0.01373	SMC	*SLC16A3* (1)	Altitude adaptation	[88]
19: 35.10-35.14	0.00442	SHF	*GID4* (1), *ATPAF2* (2)	Milk protein	[124]
20: 71.49-71.80	0.00272	SHF	*CEP72* (3), *SLC9A3* (2)	Feed efficiency	[125]
20: 71.65-71.80	0.00498	SRP	*SLC9A3* (2), *EXOC3* (3)	Feed efficiency	[125]
24: 49.90-49.94	0.00205	FNC	*ACAA2* (1)	Milk content	[126]
				RFI	[72]
26: 22.21-23.02	0.00021	SHF	*ELOVL3* (1)	Milk content	[127]
			*NFKB2* (8)	Milk content	[128]

q-value: q-value of the most significant SNP within the significant genomic region

Breed: breed names are shown in abbreviated form (full names are in Table 1)

Candidate genes: candidate genes within significant genomic regions

*RFI* Residual feed intake

The full list of genes is in Additional file 2

## Discussion

Both the economic and fitness-related traits that were highlighted in the current study are quantitative traits, which are generally affected by many genes, most of which have small effects. Selection for complex traits may occur simultaneously across many loci (with less intensity), which would leave weak signals across the genome [35]. In spite of this, selection for polygenic traits with some alleles that have a large effect may leave detectable signals. In the current study, genomic regions that were under putative selection in five native and commercial Swedish dairy cattle breeds from different parts of the country were studied. Selection signatures were identified by decomposition of *p*-values of five test statistics, instead of using single statistical tests separately or focusing on regions detected in more than one test. According to previous studies, composite measures of signatures of selection can provide an unbiased criterion to identify variants under selection more precisely [15, 36]. Therefore, by using decomposition of *p*-values to identify signatures of selection, candidate genes can be identified with higher power and greater precision for future research in medicine, agriculture, and livestock breeding.

The clear clustering of breeds into two groups (mountain breeds vs. middle–southern breeds) as shown in the PC plot presented here was not in complete accordance with the results of Upadhyay et al. [16] who could not clearly differentiate all the Swedish cattle breeds in separate clusters based on the first PC. This could be attributed to the fact that a larger number of Swedish breeds, i.e. representing an additional source of variation, were included in their study [16]. In addition, a recent study showed that the Swedish Fjäll cattle are closely related to the Northern, Western and Eastern Finn cattle and Icelandic cattle [37]. However, the map of signatures of selection reported here is in line with previous studies [16, 38, 39] that showed that Swedish Red Polled and Fjäll are two distinct breeds sharing no close genetic relationship with each other.

The historical background and usage differ between Swedish cattle breeds. Sweden spans about 1572 km from north to south [40] and climatic conditions for dairy production range from the subarctic cold climate in the more mountainous north, to the continental climate in the more densely populated southern part [41]. According to our results, none of the detected regions with identified genes was significant in all the five breeds analyzed. However, significant genomic regions harboring genes were associated with the polled phenotype, body size and stature, resistance to gastrointestinal nematodes, response to hypoxia, growth and feed intake, feed efficiency, and reproduction overlapped between some of the breeds [see Additional file 3: Table S3]. The limited overlap between the detected putative regions across these Swedish cattle breeds suggest that some breed-specific selection occurred in the past. Nevertheless, in our study of signatures of selection, we found several genomic regions and genes that are involved in economic traits, such as milk production, growth, feed efficiency, reproduction and cold acclimation and that can be used for mapping causal mutations, identifying candidate genes and making better selection decisions in future cattle genetic improvement programs.

### Signatures of potential human-mediated selection

In the commercial breeds, which originate from southern Sweden, such as the Swedish Red and the Swedish Holstein–Friesian, production of large quantities of milk has been economically advantageous. The old Swedish Friesian cattle that was rather a dual purpose breed was gradually transformed into the Swedish Holstein cattle from the 1970s to the mid-1990s, when imported Holstein bulls were used to improve milk yield and udder conformation [42]. Thus, the Swedish Holstein–Friesian became a more distinct dairy breed type, with a tall and lean conformation. Interestingly in our study, genes that are known to be associated with these traits were identified for this breed. The most significant peak identified by DCMS was located on BTA14 in the Swedish Holstein–Friesian breed (BTA14:24.42-25.11; Mb; q-value (most significant SNP) = 2.2E − 10). Previous studies have reported that the regions around this location contain numerous genes and QTL that affect cattle stature and related traits [43–47]. The genes that we identified within this region, including *PLAG1*, *CHCHD7*, *MOS*, *RPS20*, and *LYN*, are known as major genes with a role in both human height and cattle stature [44, 46, 48–52]. This region (i.e. BTA14:24.4-25.4) is also known to affect a variety of reproduction-related traits such as maturity index, scrotal circumference, age at puberty in Brahman cattle [53, 54], and blood levels of IGF1 in Brahman and Tropical Composite cattle [55]. The *PLAG1* gene is significantly correlated with the lactation phenotype [56], which suggests that this region has a major pleiotropic effect in various cattle breeds. Other genes were also identified such as *GBP4*, *FGF6*, *TIGAR*, *CCND2* (involved in body weight and stature), *GID4*, *ATPAF2*, *ELOVL3*, *NFKB2* (involved in milk yield and components), *TMEM68*, *TGS*, *LYN*, *CEP72*, *SLC9A3* (involved in growth and feed efficiency) (see Table 3).

The modern Swedish Red Cattle is a dairy breed, but historically it was important for beef production in Sweden, and still shows today a better average carcass conformation classification and a slightly higher carcass fat content than Swedish Holstein [57]. The Swedish Red Cattle breed is also known for producing higher concentrations of milk fat and protein than Swedish Holstein [58]. Our results also highlight putative signatures of selection in regions on BTA13 and 14 related to carcass quality and milk composition in Swedish Red Cattle. One of the interesting genes under putative selection is *CRH* (top ranked gene in the DCMS analysis), which is consistent with the breeding history of this breed. *CRH*, which is located at 31.49 Mb on BTA14, plays an important role in several physiological and biological pathways regarding the stimulation of ACTH (adrenocorticotropin) secretion, that up-regulates cortisol [59]. The cortisol hormone stimulates gluconeogenesis in liver and lipolysis in adipose tissue, and inhibits glucose consumption in adipose tissue and muscle [59]. Polymorphisms in the *CRH* gene are associated with marbling score [59], growth and carcass yield [60], and milk production [61, 62], which suggests a pleiotropic effect of this gene. *FOXA2* is another identified gene that is known to be one of the important transcriptional activators and plays a role in the regulation of energy homeostasis and feeding. *FOXA2* is significantly associated with chest girth, body weight, and growth traits in Chinese cattle [63]. Other interesting genes were identified in Swedish Red Cattle such as *PLAG1*, *XKR4*, and *IMPAD1*, which are positional candidate genes for pleiotropic QTL for growth traits [64]. According to the literature, *PLAG1* is associated with carcass weight, stature, body weight and milk yield [45, 46, 65–68], *XKR4* with birth weight, growth, and feed intake [51, 67, 69], and *IMPAD1* with carcass weight, stature and body weight [56, 65].

In native breeds from remote mountain areas in northern Sweden, which are located far from commercial dairies, the production of milk suitable for storable dairy products such as cheese was most valuable in the past. A putative signature of selection was identified in breeds from the northern region (i.e. Fjäll and Fjällnära) containing the first ranked gene, i.e. *FCHSD2* and the gene *AQP7* in the Fjäll breed. A meta-analysis on French dairy cattle breeds reported *AQP7* and *FCHSD2* as candidate genes within QTL regions on BTA8 and 15, respectively, associated with milk fat percentage [70]. We also identified two other genes (*UCP2* and *UCP3*) in the Fjällnära breed, for which significant associations were reported between a polymorphism in *UCP2* and calving interval in dairy cattle, and between polymorphisms in *UCP3* and production and fat content in dairy cattle [71] and carcass phenotype in beef cattle [72, 73]. Gene expression analyses also revealed an association between *UCP2* and residual feed intake in Nellore cattle [74].

### Body size and cold acclimation

Compared to the commercial southern breeds, and in addition to selection pressures for milk yield and composition, historically the native breeds had to cope with cold weather, in some high mountain regions, a limited amount of feed, and even starvation during winter [75]. These breeds generally have a smaller body size (see Table 1). The DCMS analyses identified potential evidence of adaptation to the cold climate of the mountain regions characterized by a very limited amount of feed supply (see Table 1). We identified several genes including *CCND2*, *FGF6*, *TIGAR*, *KCNA6*, *NDUFA9*, *AKAP3*, *C5H12orf4*, and *RAD51AP1* on BTA5 (BTA5: 105.75-106.52) that have been under strong selection (q-value 2.6E − 13) in Fjällnära cattle. Interestingly, a genome-wide association study (GWAS) on cattle populations from Siberia showed associations between these genes and body measurements [76]. In a gene expression study of bovine QTL, *TIGAR* was found to be significantly differentially expressed and associated with body weight [77]. Moreover, a meta-analysis of GWAS studies for cattle stature reported *CCND2* as the second most significant gene regulating stature in mammals [78]. Another gene, *CSMD3*, which encodes a transmembrane protein [79] was reported to be associated with body measurements in cattle [80].

Body size and shape vary among breeds [2, 81]. Whereas most bovine breeds from temperate regions with access to a good supply of high-quality nutrients have a larger body size, breeds that live in high altitude areas with a limited amount of feed often have a smaller body size. Our findings also agree with the Geist theory [82], according to which food availability per animal during the growing season is a determining factor for body size evolution in mammals. We hypothesized that small body size was subject to natural selection in Swedish northern breeds since it probably allowed them to develop hardiness and endurance characteristics necessary for searching food and water over long distances. This is supported by a previous study that compared the grazing pattern of a native cattle (Fjäll) with that of a commercial breed (Swedish Holstein–Friesian) and showed that the Fjäll breed explored across extensive regions with an inclination towards various types of plants [83]. In addition, shortage of stored feed during winters in years with bad harvests or extra cold and long winters occurred before the 20th century. Such historical scarcity of feed, especially towards the end of long winters when animals were kept indoors, probably favored animals with a small body weight because this is associated with lower maintenance requirements [84]. As shown in Table 1, Fjäll cattle have a small body size, which is only a bit larger than that of the Fjällnära breed. However, we did not identify genes associated with body measurements in the Fjäll breed, possibly because they have been under stronger artificial selection for milk production and kept in areas of northern Sweden with less harsh conditions.

We also identified genes under selection in the northern breeds, including *PPP1R12A*, *AQP3*, *AQP7* and *SLC16A3* in the Fjäll breed, and *UCP2*, *UCP3*, *ACAA2* and *TIGAR* in the Fjällnära breed, which are known to be related with altitude adaptation and response to hypoxia. Hypoxia or hypoxic stress is described as a decline in oxygen levels below the normal levels of 20.8 to 20.95% and results in metabolic adaptation at both the cellular and organism levels. Phosphorylation of the *PPP1R12A* (also called *MYPTI*) gene is known to increase as the level of oxygen decreases [85, 86]. In a study comparing Ethiopian sheep breeds that are adapted to different ecological regions, Edea et al. [87] identified four genes including *PPP1R12A* that showed signatures of selection likely related to high altitude adaptation. Gene expression analyses revealed *SLC16A3* as one of the significant up-regulated genes (*p*-value = 1.77E − 02) in response to high altitude adaptation of sheep fetal carotid arteries [88]. For the other northern breed, Fjällnära, we found that the four genes under putative selection, *UCP2*, *UCP3*, *ACAA2* and *TIGAR*, are involved in the response to hypoxia (GO:0001666) and other related Gene Ontologies. The genes identified by the DCMS analysis suggest that the Fjällnära and Fjäll breeds are probably adapted to the high altitude mountains. However, it should be noted that the highest mountain reaches ~ 2000 m in Sweden, and less than 2500 m in Scandinavia (Norway), and that these breeds have been maintained at lower altitudes. For example, the remaining Fjällnära cattle were sampled on farms located at 485 to 682 m above average sea level. Thus, their adaptation to high altitudes may have occurred before their recorded history in Scandinavia. A functional gene network analysis, *AQP3* and *AQP7* (and *AQP5*, another gene of the aquaporin family, which we did not identify here but was reported in a study on Russian cattle breeds by Yurchenko et al. [20]) were also identified as candidate genes for the regulation of thermal adaptation via the transport of water, glycerol, and small solutes across cell membranes [89].

### Signatures of selection for disease resistance genes

Until the end of the 19th century, the northern cattle grazed on woodlands in the summertime and had to be guarded from predators, which is why they are assumed to have developed hardiness and robustness to cope with their local environment [90, 91]. In concordance with the historical background of these breeds, our results suggest selection for stress response and immunity-related traits. Our results revealed an overlapping putative region under selection including the *FCHSD2*, *P2RY2*, *P2RY6*, *ARHGEF17* and *RELT* genes in the Fjällnära and Fjäll breeds. Moreover, the *P2RY6*, *ARHGEF17* and *RELT* genes were also identified in Swedish Red Cattle [see Additional file 3 Table S3]. *FCHSD2, RELT* and *ARHGEF17* have already been identified in sheep as candidate genes that play a role in the serum levels of immunoglobulin A (IgA), which is an indicator of resistance to gastrointestinal nematodes [92]. In addition, two other genes, *CYP4F2* and *SLC10A2*, were identified on BTA7 and 12 in Fjäll Cattle. In cattle, *CYP4F2* was reported to be involved in the host resistance to the intestinal worm *Cooperia oncophora* [93] and to gastrointestinal nematodes [94]. Hempel et al. [95] showed that *SLC10A2* was among the top five most significantly up-regulated genes (q-value = 0.000897) in a comparison between infected vs. uninfected cows with *Mycobacterium avium* subsp. *paratuberculosis* (an intracellular bacterium). Paratuberculosis infections are very rare in Sweden, but this gene could be involved in the response to infections by other bacteria or selected for other reasons related to its role in the uptake of intestinal bile acids. Our findings suggest a possible selection for adaptive immunity genes in these Swedish indigenous breeds. These genes explain a small proportion of the variance [92], which suggests that resistance/susceptibility to gastrointestinal nematodes is a complex trait that is determined by multiple groups of gene networks rather than the effect of an individual gene. However, since the *P2RY6*, *ARHGEF27*, *RELT*, *FCHSD2*, *CYP4F2* and *SLC10A2* genes appear to be under selection in the Swedish indigenous breeds, it would be interesting to analyze them in more detail. Moreover, future studies should investigate whether phenotypic variation in susceptibility to gastrointestinal nematodes or other important traits exists between these breeds.

The four *CHIA*, *CHI3L2*, *PRDM2* and *KDM5B* genes that we identified on BTA4 and 16 in the Swedish Red Cattle were previously reported as disease resistance genes (i.e. to gastrointestinal nematodes or bacterial infection) in independent studies on sheep and cattle breeds [96–99]. In Swedish Red Polled, we found that the *TMEM37* gene (BTA2: 71.46-71.66), known to be involved in the resistance to gastrointestinal nematodes, ranked as the closest gene to the most significant SNP showing a signature of selection (q-value = 0.0063). Li et al. [94] reported a set of 64 candidate genes that displayed significant overexpression at a high 5% FDR threshold level for resistance to gastrointestinal nematodes and included *TMEM37*. Similarly, we detected *SPSB1* (BTA16: 44.96-45.25), which was previously reported to be under selection in a meta-assembly of studies on signatures of selection in various cattle breeds [100], and in another analysis of bovine signatures of selection [101]. *SPSB1* (along with *SPSB2*) regulates the amount of nitric oxide (NO) produced via the induction of NO synthase [102]. Nitric oxide is one of the main contributors of reactive nitrogen known to have a major role in host defense by killing intracellular pathogens [102]. In Swedish Holstein–Friesian, the *GBP6* gene identified on BTA3 (BTA3: 53.73-55.25), is part of the six top genes that are known as key players in immune response to *Mycobacterium avium* subsp. *Paratuberculosis* [103].

A relatively small number of genes (n = 61) was identified in significant genomic regions of the Swedish Red Polled breed (Table 1). More importantly, by reviewing the literature, we found only a small number of genes in the detected selection signature region in this breed that were associated with acclimation or economic traits (Table 3). This could be attributed to the rapid reduction of N_e_ of this breed (genetic bottleneck) with only 20 cows still present in 1979 [104], which suggests that strong genetic drift may have occurred in this population, although the genetic variation in this breed increased in the last decades by the importation of red polled cattle from Norway and Finland. Schlamp et al. [8] reported that the identification of signatures of selection in small populations can be problematic, since they can be weakened by the strong genetic drift that occurs in such small populations.

## Conclusions

In total, 108 genomic regions (including 355 unique protein-coding genes) that display signatures of selection in Swedish indigenous (northern breeds) and commercial (southern origin) cattle breeds were identified by incorporating *p*-values of different statistics in a single DCMS framework. These signatures of selection are in line with the history of these breeds. Some of these signatures of selection are located in regions carrying genes associated with economic traits such as milk yield and components, and growth and carcass traits, thus describing response to human-mediated selection in commercial breeds. Other signatures of selection that were detected in regions that harbor genes for body size may be involved in the adaptation of these native breeds to the specific climate of the northern part of the country, which is characterized by cold weather and, historically, by a limited amount of feed during the winter. For example, four genes that are involved in body size traits were subject to selection in both Fjällnära (a native mountain breed with a small body size) and Swedish Holstein–Friesian (a commercial breed with a larger body size). Indeed, our results may provide background knowledge to better understand the genetic mechanisms that are involved in economic traits and adaptation to local climate. Additional high-resolution studies focusing on the putative regions of signatures of selection and using larger sample sizes and recorded phenotypes may help pinpoint the candidate genes under selection.

## Supplementary information

**Additional file 1: Table S1**. List of genomic regions under putative selection (q-value < 0.05) identified using the DCMS (De-correlated composite of multiple signals) method for each breed. The first column represents the abbreviated name for each breed, and the second column shows the number (ID) of each genomic region identified as under putative selection within each breed.

**Additional file 2: Table S2.** Full list of protein coding genes identified (using UMD3.1 genome assembly) from the genomic regions under putative selection. The 1^st^ column represents the abbreviated name for each breed, and the 6^th^ column represents the number (ID) of significant genomic region under putative selection (within each breed) and the rank of each gene within that region (i.e. the value 1 represents the closest gene to the most significant SNP within each region).

**Additional file 3: Table S3**. Mutual genes identified among breeds.

## Data Availability

The genotyping data of Swedish cattle breeds used for conducting this research were already deposited into the DRYAD public data repository. https://datadryad.org/stash/dataset/doi:10.5061/dryad.wdbrv15j4.
